# Calomel in the Army

**Published:** 1863-06

**Authors:** 


### CALOMEL IN THE ARMY.

The Surgeon General of the United States Army has issued the following very extraordinary order:

SURGEON GENERAL'S OFFICE,
Washington, D. C., May 1, 1863.

1. From the reports of medical inspectors and the sanitary reports to this office, it appears that the administration of calomel has so frequently been pushed to excess by military surgeons as to call for prompt steps by this office to correct this abuse; an abuse the melancholy effects of which, as officially reported, have exhibited themselves not only in innumerable cases of profuse salivation, but in the not infrequent occurrence of mercurial gangrene.

It seeming impossible in any other manner to properly restrict the use of this powerful agent, it is directed that it be struck from the supply-table, and that no further requisitions for this medicine be approved by medical directors. This is done with the more confidence, as modern pathology has proved the impropriety of the use of mercury in very many of those diseases in which it was formerly unfailingly administered.

2. The records of this office having conclusively proved that diseases prevalent in the army may be treated as efficiently without tartar emetic as therewith, and the fact of its remaining upon the supply-table being a tacit invitation to its use, tartar-emetic is also struck from the supply-table of the army.

No doubt can exist that more harm has resulted from the misuse of both these agents in the treatment of disease, than benefit from their proper administration.

W. A. Hammond, Surgeon General.

Mr. Editor:—The above "order" will possibly create more discussion and feeling than any one issued, from a subordinate department, during the war. It asserts, as *fact*, from "reports of medical inspectors and the sanitary reports to this office," sources of supposed knowledge and information—that which the public at large, and the medical profession will be startled at reading—without naming the department, or *who are* the "military surgeons" who have committed the "*abuse*," the public at large and the profession are required to believe, from the remedy proposed, that *every surgeon and every department* has been guilty of "abuse," and the means of preventing future evil would impress the *fact* as a *general charge* by withholding from *all* "this powerful agent." The Surgeon General by this order does not propose to correct, modify, or amend the evil—but radically to destroy the *agent* or *source* of the evil. For three thousand years the most gifted minds have been investigating nature for agencies in the cure and prevention of diseases, and the motto "*Ubi virus ibi virtus*" is now regarded as an axiomatic fact. The *potentive agency* of any remedy has never before been regarded with disfavor—on the contrary, pharmacy, for fifty years, has busied its votaries with the *one* and most desirable demand—*potency*, *dynamia*, *concentration.* Discard from every remedy effete ingredients--Morphia, Quina, Strychnia, Hyd. Acid, &c., is the result and boast of the research. No educated surgeon would discard a remedy because of its powers—for he knows its usefulness consists in its *power*. The risibility of an educated man is excited by the quintillionth part of a grain of charcoal from the want of *potency*. Why then should his fears be alarmed when dealing with agencies, whose power he can calculate, and over whose power he has complete control? This education and acquisition of knowledge makes the distinction in life and qualifies men for stations that all other guards would render dangerous to themselves and others. Why educate physicians, engineers, pilots, &c., &c., if it be for any other purpose than to acquaint them with powerful agents, and so to restrain and subdue those agents as to make them subservient to man's advantage? Electricity is a "powerful agent," yet it is guided on wires and made to "tick out" news of great and startling import. Steam, the most "powerful agent," has added to man's happiness, and extended blessings with a more liberal hand than any discovery of either ancient or modern times, yet its *power* is its utility, and by education and restraint this "powerful agent" is made man's servant and greatest benefactor. I will not pursue this subject, as its full elimination would strike from the list of remedies Opium, Chloroform, Digitalis, Veratria, Quinine, and a host of most "powerful agents," and leave us only such as from their impotency would produce neither results for good nor evil. I can not conceive the Surgeon General meant that Calomel and Tart. Ant. were the only "powerful agents," and consequently struck them from the list, but that they were powerful and therefore should be stricken off. Why should others of equal power be retained? Because they are useful, would be the reply. In what consists their usefulness? Their *power*, because if *powerless* they would be *useless*. Ergo, all "powerful agents" should be stricken from the supply table. I would respectively assert that Tinct. Ver. Viride is more *dangerous* because more *pow-*

*erful* than either Calomel or Tart. Ant., and a use of the remedy will illustrate this assertion.

In all our Western States an Examining Board was instituted by law, and is now in session daily exami ning candidates. I know several of these Boards and was examined myself. All these Boards are men of equal medical and surgical knowledge, and in two Boards with whom I am acquainted are men not surpassed in acquirements in this or any other country. These men are competent, honest and conscientious—and to charge the military surgeons indirectly or directly with ignorance and abuse of the use of "powerful remedies" is to censure the action of these Boards. That there are men in the army equal to those in private practice is not saying too much—that ignorance and abuse of any remedy to any extent exists in the army is saying what neither the "*medical inspectors*" *nor the sanitary reports* can prove. It but accords with the reports of the use and confiscation by Army Surgeons of food and clothes sent by the public for the use of the sick. Few months since, every newspaper was full of reports of these "rascalities," and no men since the war have so liberally shared public-scandal and falsehood as the surgeons. Selected from the very highest positions in society—men of acknowledged qualifications, they have borne more calumny and suffered more in personal and professional reputation than any other department. The public are too prone to believe the statements of bad men, and the pale and emaciated returned soldier in his recounting of his escapes and suffering never fails to enter a deep and damning record against the surgeon, whose skill has saved his life, and whose only fault was not giving him what was not supplied; or withholding from a diseased and capricious appetite that which would have destroyed his life. These are the sources of abuse—and moreover, in the anxiety to do good and in the ignorance of what way to consummate the desire, the sanitary visitors find in all hospitals and camps, a man who, while he appreciates the motives, knows the results and interdicts officious interference.

After the battle of Shiloh, a number of good men and good women visited the camps—many, very many, God bless them, were efficient, patient and useful—many others knew too much for a nurse and not enough for a surgeon and returned in ineffable disgust, criticising every thing, from the manner in which the battle was fought to the treatment of the wounded, and, in some instances, assumed the control of boats, hospitals and tents, to the exclusion of all outsiders.

From such sources the Surgeon General obtained his information. Of this class was not the Governor of this State. He came, took off his coat, asked for work, took advice and followed it, and has acquired a reputation, and deservedly so, that will outlive his highest official actions. If he found any thing deficient, or wrong, he supplied the deficiency and corrected the wrong. Another, and from an equally unqualified a source as these, in the Western and Mississippi Department was from Medical Inspectors—or what is of the same class, Brigade Surgeons, Medical Directors, &c., &c. I saw fifteen during my stay in the army; thirteen of these were from north of the Connecticut river. They were sent to manage the surgeons of the Mississippi. Not one of these thirteen had seen a case of intermittent fever generated in the locality where they practised, as no case of intermittent has occurred north of Connecticut river during their natural lives. These reports to the Surgeon General are made by men whose knowledge of western diseases was acquired from daily visits to camps, and if in them some abuse of Calomel were found (and that there were such cases there can be no doubt), these cases were magnified until a "pressure" too great for the shoulders of the Surgeon General was brought to bear and in an unguarded moment, he has perpetrated a slander on his profession, and has arraigned indirectly men who are his peers in the profession although they do not wear his shoulder straps.

To directly arraign the whole number of military surgeons from such sources of information is unkind and unjust, and the Surgeon General will repeal this order and make either an explanation or an apology for its issue. He begins at the wrong end—interdicts the "powerful agents," but leaves the inferential stupid apes in positions where they can obtain for a trifling sum, on private account, any quantity of these—and more—have in their hands and will use (if stupidly they use these two "powerful agents,") agents more potential than either. Why not privately address the offending parties and give them warning and a means of defense—rather than sweepingly include all. I have lived through two medical reforms and was seeing the rapid disappearance of another less dangerous, because more harmless, but never before has come from the head of the profession (or at least he should be) so sweeping a charge for the enemies of legitimate medicine as this order.* He demands effects without causes—brick without straw—responsibility—yet cripples resources. It certainly means no surgeon shall use Calomel or Tart. Ant., or why strike them from the supply? If, therefore, any one, in the treatment of Syphilis, should use Calomel—or in Pneumonia, Tart. Ant.—he disobeys the spirit of this order, and would be liable to censure. Should, however, the case of Syphilis, which all know to be amenable to Calomel, terminate in secondary and fatal symptoms—should the Pneumonia from the first stage of congestion and inflammation run to the second one of hepatization, and the third of softening, and Tart. Ant. (who all know, in and out of the army,) could have controlled it in its first stage, be withheld, who is responsible? Surely not the army surgeon, as he has not had the remedy furnished, and in its denial, interdicted its use. As a friend of the Army, as the friend of the Army Surgeon, and the friend of the Surgeon General, we say, let no such interdiction exist. Give all means for combatting disease and hold men responsible. These acts are as reasonable as the

* This morning, a noted Thompsonian said, in my preseuce, "that Sam'l. Thompson was vindicated in his opposition to *Mercury* by the highest American authority." A little pill doctor in this city is willing to bet a hat "in sixty days an order will be issued to restrain the use of a number of *Allopathic remedies* and the introduction of Homœopathic practice."

man who refused to allow his son to learn to write because he might commit forgery.

Had this order emanated from General Hascall, we would not have been surprised, but coming from one appointed over his seniors—one who had taught his profession, and one in whose hands the honor and fame of its usefulness are placed—it is strange that some other mode was not used to remedy an alleged evil than the degradation and insult of all the surgeons in the army.

That something will be said of this order in the coming U. S. Medical Convention, I do not entertain a doubt. We look to this body for protection, and if I mistake not the temper and character of its members, there will be a settlement of this and several other evils under which the profession has labored during this war—until then *nous verrons*.

I will resume this subject in your next number if the order be not withdrawn. Yours,

A SURGEON.

